# The Shifting Public Health Landscape and Virtual Learning Environment: The Effectiveness of Practice-Based Teaching Delivered In-Person, Virtual, and Hybrid

**DOI:** 10.3390/ijerph20042867

**Published:** 2023-02-06

**Authors:** Stacey Cunnington, Alyson Codner, Eva Nelson, Donna McGrath, Jacey A. Greece

**Affiliations:** 1 Department of Community Health Sciences, Boston University School of Public Health, 801 Massachusetts Avenue, Boston, MA 02118, USA; 2 Educational Policy and Planning, Boston University School of Education, Boston, MA 02118, USA

**Keywords:** practice-based teaching, virtual learning, public health workforce, public health education, competency achievement

## Abstract

The pandemic necessitated teaching competencies that allow public health (PH) students to be immediately workforce ready. The shift to virtual learning provided an ideal time to consider pedagogies focused on applied learning opportunities, such as practice-based teaching (PBT). This multi-year, post-test evaluation of one PBT course explored differences in students’ competency achievement immediately post-course with different modalities of delivery: fall 2019 in-person (*n* = 16), summer 2020 virtual (*n* = 8), and fall 2020 hybrid (*n* = 15). Using a variety of methods to assess across semesters, the study found virtual and hybrid learning environments resulted in equally high levels of competency achievement as in-person delivery. Regardless of course delivery, students reported, with no difference across semesters, PBT directly contributed to their workforce readiness, helped with acquisition of essential workforce skills such as problem-solving, leadership, and teamwork, and led to skill and knowledge acquisition they would not have achieved in a non-PBT course. The increased emphasis on virtual learning changed the higher education landscape and the need for students to be workforce-ready with the technical and professional skills demanded by the field and offered opportunity to redesign courses with an emphasis on applied opportunities. Virtually delivered PBT is an effective, adaptable, and sustainable pedagogy worth the investment.

## 1. Introduction

The outbreak of COVID-19 amplified the need for the public health (PH) system to respond immediately with a well-trained, adaptable, and ready workforce. The pandemic created challenges for not only public health in general, but for higher education as well. The public health sector in general faced funding and staffing challenges, most notably through a lack of dedicated pandemic funding for local public health and insufficient staffing to respond to the myriad of issues resulting from the pandemic [1]. Other challenges faced by the public health sector prompted by the pandemic include inconsistent, inadequate guidelines and communication from federal and state governments [1,2] and a lack of data tracking and engaged community partnerships. In response to these challenges, academic public health responded by deploying public health masters and doctoral students to understaffed local health departments to assist with COVID-19-related efforts [3,4,5], similar to graduating medical and nursing students early to serve the demand of the front lines. This response to the changing demands of the public health sector placed increased pressure on public health education, requiring rigorous training and a heightened level of workforce readiness so graduates could immediately integrate into the existing system and successfully apply their skills.

The pandemic highlighted that serving the ever-changing field of public health has always required a workforce capable of immediately applying tangible skills across content areas [6,7,8,9,10]. Accreditation standards have consistently demanded that schools of public health (SPH) ground professional public health education in real-world situations to achieve this [10]. Due to the pandemic, the necessity of teaching skill-based competencies that allow students immediate hands-on experience so they can enter the workforce prepared to meet challenges [11] became even more relevant. Given the current pandemic and considering the changing employment sectors of MPH students [12], teaching effectively with technology has never been more essential to learning [13].

Practice-based teaching (PBT) is a pedagogy long touted for its benefits but not often implemented for its perceived resource-intensive nature [14]. PBT combines traditional student learning with practice-based opportunities through work on a current problem for an agency and the community it serves [15]. PBT courses can be effective when planned, implemented, and evaluated according to a specific framework, such as PBT STEPS [15,16], and they offer benefits to all stakeholders: the student, SPH, the agency, the community the agency serves, and alumni [16,17].

PBT courses in public health and across disciplines, like traditional lecture-based courses, adhere to course learning objectives, assignment guidelines, and a syllabus to ensure students acquire the knowledge and skills to meet the requirements of the course. PBT courses contain some didactic components; however, the emphasis of time is placed on direct application of concepts both within the classroom setting and outside the classroom with discussion about the application, the interactions, meetings, and brainstorms with collaborating agencies and their stakeholders throughout the semester; the assignments that are intended for use in the field and therefore undergo numerous rounds of internal review and feedback; and, the consultations with the teaching team to tailor general course content to the specific project of focus with a number of team-building and sharing opportunities [15]. In a PBT course, the learning happens from working on the problem presented to the students (as experienced by the field) versus the learning being a prerequisite of addressing the problem.

Although in-person delivery of PBT was used in SPH before the pandemic began, the pandemic forced use of different and innovative delivery modalities of PBT to engage both students and collaborating agencies. The virtual and hybrid modalities used during the pandemic allowed students to gain experience in the field during a time where students struggled to access these opportunities and prompted engagement with agencies that were outside the geographic bounds of the SPH. Given that faculty at SPH have been challenged to find applied opportunities for students to implement competencies delivered in the classroom to the field during the pandemic, the use of PBT emerges as a possible solution. Evaluations of learning delivered through different in-person and virtual mechanisms have shown equally effective learning outcomes [18], and as such, SPH accreditation guidelines have proposed updates to reflect virtual learning opportunities [15].

PBT courses support a variety of learning styles [19], prepare students with essential workplace skills, provide benefit to collaborating agencies and communities [20,21], and can be adapted to online delivery, which is especially necessary given recent increased emphasis on virtual learning [22]. Even with these benefits, there are challenges that have hindered adoption and implementation of the pedagogy including substantial work outside of class for students imposing schedule constraints [23]. PBT, which is often used in clinical training such as in nursing and medicine where students can immediately apply the information in a practical setting, has in recent years extended into other disciplines where students can apply their knowledge and skills to a real-life setting and problem [24]. Assessing the benefits and outcomes of PBT is essential given new research on the long-term effectiveness of PBT in the accomplishment of technical and professional competencies, the positive outcomes of PBT reported by collaborating agencies, [25,26] and the promise of PBT for both in-person and virtual course delivery.

### 1.1. Overcoming Barriers to Practice-Based Teaching

**Figure 1:**
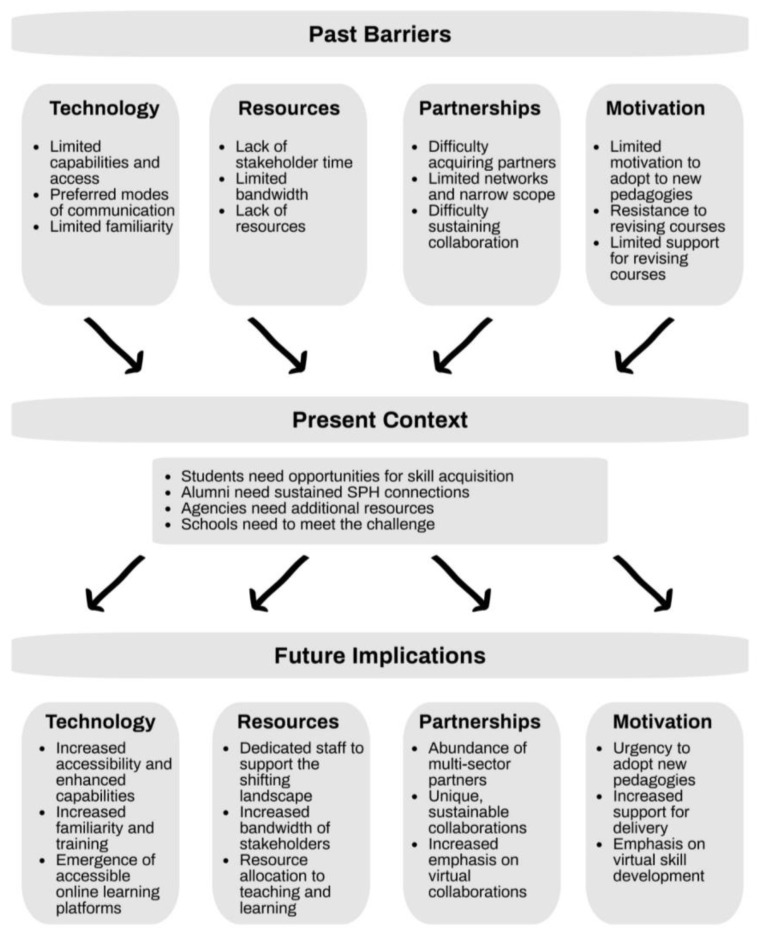
Factors for Virtual, Scalable, and Sustainable Practice-Based Teaching.

Learning by doing is the best way to acquire skills, engage in a topic, and prepare students for job success. Faculty must create these opportunities and SPH must support this endeavor. In the past, before the pandemic, there have been barriers to shifting from traditional courses to PBT, including lack of resources, limited access to technology, difficulty securing partnerships, and resistance to change. Many of these barriers to PBT, however, are in the past because of increased access to technology, reallocation of SPH resources and supports to redesign courses, and an urgent need for the field to utilize a growing public health workforce. Given the pandemic, the cost of implementing PBT is now, in the present context of still managing the pandemic, more than ever far outweighed by the benefits (Figure 1).

One argument against transitioning traditional courses to PBT is the time required before and during the semester to develop, implement, and participate in a PBT course [23]. In addition, students’ personal, school, and job commitments can impose restrictions on time and availability to fully commit to a PBT course [25]. Similarly, the limited resources of many agencies can also impact engagement and responsiveness during the semester [20]. The new norm of virtual collaborations may streamline efforts to engage in meaningful ways for PBT. For example, existing and new resources earmarked for adapting teaching and technology to the shifting higher education landscape can result in newly created websites to establish the initial collaboration. Online platforms and learning management systems during the semester enhance and sustain the partnerships with the agencies and are resources that are now commonplace in virtual learning with schools and agencies investing in technologies and opportunities to interact virtually as part of daily operating procedures [14,27].

Due to the use of online platforms during PBT classes, lack of technology to initiate partnerships and continually engage students with collaborators during the semester is another challenge of PBT. Meaningful engagement in PBT requires access to and familiarity with technology that allows for seamless connection and collaboration [16]. Traditionally, this was difficult for SPH that had specific password-protected platforms, faculty with limited technology experience, agencies with minimal technological capabilities, and students needing access to multiple learning platforms. Due to COVID-19, many people in the U.S. are now familiar with operating remotely and, while challenges still exist, there have also been benefits to virtual participation [22]. These benefits extend to PH courses that utilize PBT, which has resulted in remote and local agencies simultaneously working together to connect with all stakeholders, regardless of geographic location, while still maintaining the high quality of course deliverables and communication.

A third barrier has been difficulty in securing partnerships. Faculty may struggle to find the right partnering agency to engage in a PBT course, thinking that it needs to be restricted to an agency with a mission that directly advances public health. This pandemic has made it clear that public health touches every sector of society. Health is a basic need [28], and as such, strategic partnerships for a PBT course can be found anywhere and the outputs of the collaboration can vary greatly. While many PBT collaborators in public health courses are health agencies, their primary partners represent law enforcement, social work, hospital administration, and businesses, among others. All these partners benefit from the students’ final deliverables and class resources to fill gaps in their ability to address the issues presented. The classroom partners should not be restricted as public health touches all sectors of society.

Finally, schools and faculty have been resistant to changing the status quo when the structure seems to be working or when the changes require a good deal of effort or resources without guarantees the changes will be effective. Whether we are ready or not, the current context of both PH programs and the field is motivating us to change our pedagogies to quickly produce competent students with prior field experience and who are workforce ready. Students have had to embrace technology as part of their learning platform given the restrictions from the COVID-19 pandemic and the resulting shift toward virtual learning and virtual workforce positions. Agencies have had to pivot and shift resources to meet the changing needs of the populations they serve [14,29]. Faculty have had to revise courses for maximum effectiveness delivered virtually, online, or in a hybrid setting to meet a shifting demand for virtual and distance learning [23]. SPH have had to recognize that pedagogies that previously worked may no longer suffice in post-COVID-19 times. All stakeholders have had to evolve and adapt to find innovative, cost-effective, and sustainable solutions to pressing problems in real time. This is the hallmark of PBT courses, which results in deliverables that are targeted, implementable, and innovative and partnerships that are sustainable and impactful.

### 1.2. The Benefits to Implementing Practice-Based Teaching

There is an increased demand for prepared, experienced public health professionals in response to the changing demands of a field that is trying to serve communities’ evolving needs. As a result, there is an unprecedented urgency to adjust the way public health academic programs are facilitated to accommodate for all types of learning, whether in-person, virtual, or hybrid. While there has been a shift back to traditional ways of delivering courses, adapting to a new terrain of virtual learning and determining skills-based curricula moves beyond pedagogies that require solely in-person interactions and welcomes the scalable application of PBT in SPH, even with the commonly cited past barriers to implementing it. This results in tangible benefits for all stakeholders both in the immediate and long-term landscape with agencies from PBT courses reporting immediate and sustained benefit from the collaboration [26].

**Figure 2:**
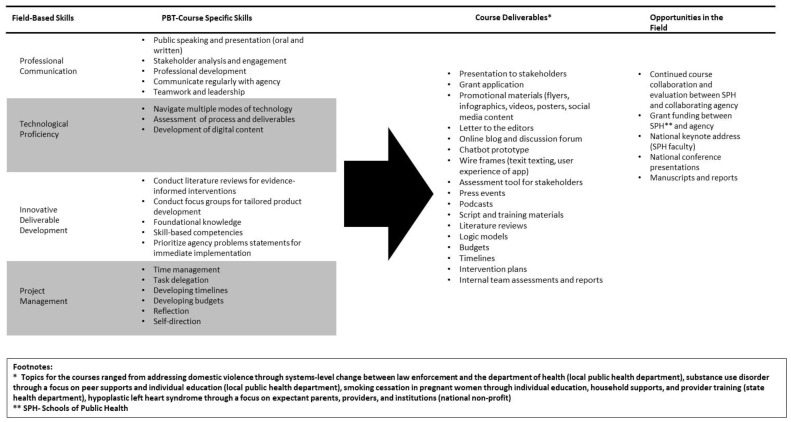
Outcomes of a Practice-Based Teaching (PBT) Course—Student Skill Acquisition, Collaborating Agency Deliverables, and Public Health Impacts.

Given the urgency for experienced PH professionals felt in response to COVID-19, students are seeking educational opportunities that allow maximum skill acquisition (Figure 2) regardless of mode of course delivery and that allow exploration of where they fit into the system [30]. SPH that utilize PBT allow students to obtain skills far beyond what a traditional course teaches, which gives them the confidence to innovate and take risks to develop sustainable solutions, demonstrates how they can immediately serve the field, and exposes them to relevant issues that may be outside their interest or region but that require real effort to solve. It also gives a space for students to try new ideas and innovations and collaborating agencies a space to learn new approaches. PBT creates opportunities and connections between students and agencies, thereby streamlining the entrance of trained PH graduates into the field.

The ultimate goal of PBT courses is to prepare students for success once they graduate and enter the field. Examining the value of PBT for alumni helps demonstrate the effectiveness of the modality. Alumni engagement with PBT reinforces their connection to SPH, allows them connection to and recruitment of current students, provides lifelong learning opportunities for skill redevelopment, and streamlines PBT course collaborations given their intimate learning experience with the school [12]. For example, SPH alumni may have a need for additional competencies to address COVID-19-related issues that were not part of their course of study but that they can access through sustained connections. Alternatively, they may have pressing problems within their agency and collaboration with a course can result in quick and cost-effective solutions. SPH often have alumni engagement as a top priority, with offices and staff dedicated to this effort, to establish networks for current students, provide lifelong learning opportunities for alumni, and foster an ongoing partnership between academia and the field. PBT is one way to facilitate this engagement and result in alumni having the ability to continually access support and resources from their SPH [20].

PBT allows collaborating agencies, which are typically under-resourced and short-staffed, access to high-quality deliverables and innovative ideas at no cost to them [20] during the semester. In addition, they have access to the faculty, students, and alumni they collaborated with after the semester. As a benefit to the student and the agency, PBT introduces the opportunity for networking. This not only benefits the student by giving them exposure to potential professional opportunities in the field, but also benefits the stakeholder and their agency by gaining access to a pipeline of experienced students who are already familiar with the stakeholder’s work. Even in non-pandemic times, the benefits to agencies of a PBT course are extensive [16], and the shift to virtual participation expands the pool of potential PBT partnerships.

Finally, SPH graduate programs have always sought to raise the standards of education through a variety of practice opportunities to expose students early and often to the ever-changing needs of the diverse field [11]. This is even more imperative now during this time of re-envisioned modes of teaching and learning to successfully deliver virtual, online, and/or hybrid courses that are interactive and flexible. PBT allows SPH to continually strengthen their ties to the communities in which students are being trained to serve and establish lasting relationships for research, practice, and further teaching.

This study aimed to explore if there are differences in competency achievement immediate post-PBT course across different teaching modalities—virtual, hybrid, and in-person. The hypothesis is that the delivery of hybrid and virtual is just as effective as delivery in person when using a framework for the design and implementation of a PBT course. The usefulness of teaching evaluations has long been debated in general, but it is particularly important for PBT courses where conventional methods of assessment do not typically measure student achievement and application [31,32,33]. The benefits to PBT have been documented in an in-person delivery model [15,26,34] with ongoing evaluations recommended [35], but to date there is limited research on the effectiveness of PBT delivered virtually or in hybrid. Given the immense benefit PBT offers coupled with the investment of time and resources to effectively implement it, understanding the benefits in a virtual environment are important to understanding how it is best delivered for adoption and expansion as the higher education landscape shifts to remote course delivery and the opportunity for virtual collaborations expands.

## 2. Methods

To examine the utility of PBT, we conducted a multi-year post-test evaluation of one course over three semesters with different modalities of delivery: fall 2019 in-person delivery, summer 2020 virtual delivery, and fall 2020 hybrid delivery. The course was designed and delivered according to a PBT framework; PBT STEPS [16] was applied by the same instructor across all three semesters, using the same syllabus and assignments. Whether in-person, virtual, or hybrid delivery was used, the course delivery, content, and assignments remained consistent. While this course is required of students in the health communication certificate and optional for students in the intervention design certificate, each semester, students from across public health disciplines enroll. The delivery mode they received in this study was dictated by the policies of the school for that semester.

The evaluation used a variety of methods to assess and compare across the semesters outside of the usual student course evaluations, as described below, to examine the course effectiveness of achieving outcomes across PBT delivery modality. The outcomes were assessed according to the course logic model [15], and the evaluation was conducted by an independent evaluator. The university’s Institutional Review Board (IRB Number #H-37484) approved all activities as an exempt study.

### 2.1. Practice-Based Teaching Framework: PBT STEPS

Absent a user-friendly and logical road map for using PBT in public health education, the pedagogy can be difficult to implement. The PBT STEPS framework helps faculty design and teach a PBT course through securing partnerships (S), technology and training (T), engagement and implementation (E), presenting deliverables (P), and sizing up results (S) [16] to yield impactful deliverables; this framework provides a road map for collaborating agencies to engage in successful collaborations. This framework is also adaptable to the time allocated for development of a course, as well as the time frame of course implementation. In addition, PBT STEPS is an iterative process. Each step builds on the previous step, but any step can be revisited to ensure successful application of the framework. The course evaluated in this study was designed and implemented according to the PBT STEPS framework and has for seven years been offered multiple times per year.

### 2.2. Course Design

Communication Strategies for Public Health (SB806) is an advanced intervention planning and communications course for Master of Public Health (MPH) students at the Boston University School of Public Health, a top-ranked SPH in the country. SB806 is focused on changing the health behavior of a target population that is experiencing a problem or an influential environmental agent that influences the target population of the problem. The course leads students through a step-wise planning process [36] to design an evidence-informed, innovative, and feasible intervention that directly addresses a target population’s knowledge, attitudes, behavioral skills, self-efficacy, and other individual-level determinants of behavior. The target of these efforts can be the individual experiencing the problem or some other key influencer—organizational managers, community leaders, or policymakers—to take action. Training students to be prepared for, adaptable to, and competent in all forms of intervention design and communication is essential to the changing field of public health, particularly now with the shifting priorities and emphasis on technology for effective communication.

SB806 is a PBT course in which groups of students work with a collaborating agency to develop a public health intervention and targeted communications strategy. The course is offered every fall, spring, and summer by the same instructor, a co-author of this manuscript, who is trained in program design, communication, and evaluation and who spent many years in public health consulting before coming to academia. To adhere to an unbiased evaluation, this course has been independently evaluated by an education evaluation consultant since 2014 and guided by a logic model. Across the three semesters of the course evaluation, course logistics differed depending on the delivery format of the course, but the assignments, content, learning objectives, and timeframe remained the same. The in-person delivery format met during the fall 2019 semester in a BUSPH classroom once per week for 3 h per class over a 14-week semester. The virtual delivery format course met during the summer 2020 semester, completely through Zoom with the instructor and all students on Zoom, twice per week for 3 h per class over 7 weeks (as is the structure of the summer offering at the SPH). The hybrid delivery format met during the fall 2020 semester and the instructor taught from a BUSPH classroom. Students could choose to attend in-person or virtually through Zoom, which was set up in the classroom. The course met once per week for three hours per class over a 14-week semester. Each semester had a maximum of 24 students.

Students receive a list of problem statements and collaborating agency descriptions before the first class and are assigned a group of three to five students. They prepare a series of written assignments (i.e., literature review, intervention description and detailed plan with budget, logic model, and timeline) and develop a communication strategy with specific media executions. The class concludes with delivery of an oral 30-min group presentation to introduce their plan. Students learn the course competencies (Figure 2) through assigned readings, lectures, and case study discussions, followed by skill-building exercises, with dedicated class time for group work in addition to consultations (in-person or virtual) with the agency and the course instructor, which are the hallmarks of PBT (Figure 2). In the three semesters that were included in this study, the collaborating agencies engaged with their student groups remotely.

The collaborating agencies in this class differ greatly but the commonality is that each has a priority area of concern related to the public health field, one or more target populations of focus to address the problem, and an overarching behavior-oriented goal to achieve. Across the three semesters in this study, collaborating agencies represented a state health department with a large staff, a local health department comprised of one director, and a national non-profit organization led by a volunteer board of directors. All primary points of contact at the collaborating agencies had previously worked with the course before as past collaborators or guest lecturers.

### 2.3. Data Collection and Course Competency Assessment

Evaluation of student achievement was assessed in a variety of ways during the course and post-course. The combination of quantitative and qualitative data collection and the variety of data collection approaches allowed a more thorough assessment of student competency achievement, which assessed both technical skills as outlined in the syllabus and professional skills necessary to engage in a PBT course. The study used the following qualitative data sources:

Course Documents: an exhaustive list of course documents were reviewed by the evaluator, accessed through the learning management system, and/or provided by the instructor to provide context to the course and for development of more targeted surveys and a deeper understanding of the course. These documents also serve as the foundation for the agency collaboration and rules of engagement for the semester.

Group Evaluations: students complete peer evaluations at multiple timepoints throughout the semester to assess the quality of their groups’ interactions, successes and challenges in teamwork and communication, and areas for improvement. Each student provides a self-assessment and overall assessment of each group member.

The study used the following quantitative data sources:

Post-Course Surveys: students completed online post-course (administered the last week of the course) surveys that were implemented in addition to the standardized student course evaluation used by the SPH. The course-specific post-course survey was constructed according to the logic model and after thorough review of the course content and goals. The post-course surveys were administered to students to assess achievement of competencies, satisfaction, and perceptions of PBT. Agreement with statements were assessed using a five-point Likert scale, with the scale ranging from strongest disagreement to strongest agreement or not important to extremely important, depending on the question stem. Post-course surveys were administered by the independent evaluator via email to students in their last class in all three cohorts: fall 2019 semester (in-person delivery, *n* = 19), summer 2020 semester (virtual delivery, *n* = 8), and fall 2020 semester (hybrid delivery, *n* = 17). Response rates were high in all three semesters.

Grading Rubrics: given the consistency of the assignments and subsequent deliverables to the collaborating agency, the same grading rubrics were applied to the three main assignments and the final presentations across the three semesters. Grading rubrics were assessed to determine the range of grades in each semester and the consistency of performance.

Finally, the study used Student Course Evaluations as a combined qualitative and quantitative data source; the SPH administers student course evaluations for every class at the end of each semester that ask for student reflections on the course, instructor, assignments, and activities. The student course evaluations contain both close-ended and open-ended questions in addition to ratings scales to evaluate the course. These evaluations are made public to the SPH community approximately two months after the course. The student course evaluations were assessed across the three semesters to determine whether there were differences in the course quality and satisfaction.

### 2.4. Data Analysis

Quantitative survey data was analyzed using SAS software (v.9) (SAS Institute Inc., Cary, NC, USA). Likert-scale questions were examined using proportions and continuous variables were examined using means and standard deviations. Post-course means were obtained from the five-point Likert-scale questions to examine differences in post-course responses across the three semesters. Given in-person learning was the traditional form of teaching, post-test scores compared hybrid to in-person learning and virtual to in-person learning. Significant differences in post-test responses were assessed using the Whitney–Mann Wilcoxon test. Sample sizes, means, and standard deviations are reported, as are *p*-values where appropriate. Qualitative data was analyzed by two coders to assess for themes in open-ended responses and feedback forms.

## 3. Results

There were 18 students enrolled in the fall 2019 in-person with 16 completed surveys (88.9% response rate), 8 students enrolled in the summer 2020 virtual with 8 completed surveys (100% response rate), and 17 students enrolled in the fall 2020 hybrid with 15 completed surveys (88.2% response rate). Of the students responding to the post-course survey (*n* = 39) in all three courses, most were female (87.5%, 100%, and 80%, respectively) with a higher proportion White/Caucasian in the fall 2019 and 2020 semesters (62.5% and 40%, respectively) compared to the summer semester (0%) and under age 25 (62.5% and 73.3%, respectively, compared to 37.5%) (Table 1).

At the post-test assessment (Table 2), students reported that PBT was important in acquiring a variety of skills that were the focus of the course though the degree of importance differed for skills and also on the semester of PBT delivery. Students were asked on a five-point Likert scale (1 = not at all important; 2 = somewhat unimportant; 3 = neither important nor unimportant; 4 = somewhat important; 5 = very important) how important PBT was in acquiring the skill. The cohort in the virtual semester rated the importance of PBT for skill acquisition higher than the in-person and hybrid semesters with all but one skill (literature reviews) rated significantly higher in the virtual semester compared to the in-person semester. The in-person and hybrid semesters had similar and not-significantly different ratings on importance of all skills listed. While PBT was important overall in skill acquisition across semesters, it was especially important in the virtual semester compared to the in-person semester with many of the skills rated significantly higher compared to the in-person semester (Table 2).

Additionally, students were asked at post-course to reflect on the utility and value of PBT. Across all three semesters, the taught in-person, virtual, and hybrid students agreed that the time invested in taking a PBT course was worth it (4.93, 4.75, and 4.67, respectively), that more MPH courses should use PBT (4.81, 4.63, and 4.64, respectively), and that they appreciated the utility of the PBT course more at the end than when they had initially enrolled (4.25, 3.75, and 4.67, respectively) (Table 3). Students across all three semesters disagreed that they would have gained the same knowledge (1.67, 2.38, and 2.0, respectively) and the same skills (1.6, 2.25, and 1.8, respectively) if the course had been taught using traditional teaching methods rather than PBT. None of these ratings were significantly different for virtual delivery or hybrid delivery versus in-person delivery. These results emphasize that whether PBT is utilized in-person, virtually, or hybrid, students appreciate the utility of a PBT course and value its use over traditional teaching methods.

The degree to which working with a client in a PBT course impacted their workforce readiness was assessed through a series of questions (Table 4). Not only do students value the skills obtained through PBT in the workplace, but they believe PBT has directly contributed to securing a position in the field. Students across the three semesters consistently agreed or strongly agreed that PBT made them more marketable, helped clarify future plans, made them better prepared to enter the workforce, enhanced their appreciation for the field of public health, and resulted in professional networking opportunities they may not have encountered otherwise. In addition, students consistently agreed that PBT helped with skill development that is essential in the workforce including problem-solving skills, leadership skills, skills working with a team, and skills they could implement in their job. These perceptions on workforce readiness were not significantly different across semesters, with the exception of students in the virtual semester agreeing to a lesser extent than students in the in-person semester that it resulted in networking opportunities they otherwise would not have encountered.

Students in the two semesters that had a virtual learning component were asked whether the virtual environment affected their development of skills and workforce readiness (Table 5). A score of three on the five-point Likert scale indicates the statement was “not impacted” by being in a virtual environment. In both semesters, students largely reported that the virtual learning environment did not impact their development of skills or workforce readiness, including problem-solving skills, leadership skills, skills they could implement in their job, skills working with a team, and they were prepared to enter the workforce. Additionally, students reported that the virtual learning environment did not impact positive experiences with the course itself, meeting course expectations/learning outcomes, producing quality assignments, engaging with course activities (lectures, brainstorming sessions, and skill-building work), interacting productively with the teaching team, establishing a personal connection with the teaching team, interacting productively with their client, establishing a personal connection with their client, establishing a personal connection with other students in the course, and staying motivated to produce their best work. The only skill significantly impacted by the virtual learning environment compared to hybrid was the development of problem-solving skills (4.25 and 3.47, *p* = 0.0232).

In addition to the post-course survey, other course materials were evaluated across the three semesters to understand student performance. In the course, there are three assignments with opportunities for draft review prior to submission. A review of the grading rubrics found that assignment grades were consistent, ranging from 85 to 96, with the first assignment receiving lower grades and grades getting higher with each subsequent main assignment. This grading trajectory is typical for this course and student performance was consistent with in-person, virtual, or hybrid instruction.

Aligning with the three assignments, students complete peer evaluation forms throughout the semester to evaluate themselves, the individual members of their group, and the quality of their group experiences. Group evaluations (*n* = 39) found no qualitative difference in how groups collaborated across the three semesters (four groups in fall 2019 and 2020; three groups in summer 2020) or the level of quality collaboration that existed. Themes that emerged included that the group experience enhanced teamwork skills and allowed for personal connections with classmates but also resulted in challenges around expectations with communication response time, internal team deadlines, and competing priorities in scheduling and work. Students reported that working in groups virtually allowed for ease of finding times to meet and streamlined the sharing of drafts and ideas, but that not having a set in-person meeting time sometimes created challenges with scheduling and adhering to internal deadlines. Quality and productivity of group meetings was not found to be affected when held virtually versus in person.

### Student Course Evaluations

SPH student course evaluations were reviewed to collect student reflections on the course, instructor, assignments, and activities. These were assessed across the three semesters to determine whether there were differences in the course quality and satisfaction. Student course evaluations were consistently high across the three semesters with students reporting a high level of satisfaction (out of a five-point rating scale) and assigning both the course and the instructor an exceeds expectation rating. The feedback across all three semesters was consistently that the course was worth the time they invested, though the time investment was higher compared to their other courses; there was an appreciation of working directly with a collaborating agency to achieve hands-on experience; and, it allowed them to develop more concrete skills for the workforce. Student feedback also indicated that the use of a quality online platform to engage with each other and their collaborating agencies was essential to success, with students in the virtual semester commenting more heavily on this and concluding that the wrong platform would make engagement in the course much more challenging.

## 4. Discussion

This evaluation of PBT delivered over three semesters of an MPH intervention and communication course found that the virtual and hybrid learning environment resulted in equally high levels of competency achievement in technical skills than in-person delivery. Students across all three semesters reported the importance of PBT as a pedagogy to acquire the skills of focus in the course, with students in the virtual semester reporting a significantly higher importance of PBT to learn the skills compared to the in-person semester.

Positive perceptions of the utility and value of PBT were high across the three semesters with students reporting that it was worth taking a PBT course even given the resource-intensive nature, that more MPH courses should use PBT, and that they appreciated the utility of the course more upon reflection than when they initially enrolled. Students also reported that they would not have gained the same skills or same knowledge in a traditionally taught course not using PBT. In addition, regardless of course delivery, students reported that PBT directly contributed to their workforce readiness and, in particular, helped make them more marketable, helped clarify future plans, prepared them for the workforce, enhanced their appreciation for the field, and provided networking opportunities they would not otherwise have. They also reported that the PBT course helped with the acquisition of essential workforce skills such as problem-solving skills, leadership skills, working with a team, and skills they could implement in their job. The mode of course delivery (i.e., in-person, virtual, or hybrid) did not significantly differ across the semesters.

Our findings are consistent with other research that shows students in PBT courses, regardless of mode of delivery, demonstrate the achievement of learning objectives and acquisition of skills during the course and attribute those gains to PBT [25]. The benefits are not just unique to students, and while this study did not explore the benefits to agencies across delivery modes, previous research has shown that there are both short- and long-term benefits to collaborating agencies when engaged in PBT, including resource and cost savings, more targeted programs for communities, and better achievement of community outcomes [23,37,38]. Faculty, who have reported barriers to PBT [19], also recognize the benefits and utility of this pedagogy [26,39], and SPH acknowledge the benefits of this pedagogy while also highlighting barriers to larger-scale adoption of it [26].

The effectiveness of the pedagogy, regardless of delivery, is evidenced by perceptions of utility and high achievement by students, as well as perceived utility by agencies and faculty both in this study and in others. The evidence of effectiveness puts many of the previously reported barriers on time and resources into perspective [20,23,25] and provides evidence that the cost is outweighed by the benefits [20]. The barriers that prevented more wide-scale use of PBT in the past are less relevant now given the changes that the pandemic has had on the way education is delivered. Considering the current health climate and the role COVID-19 has played on SPH, PBT offers an effective route to best prepare rising public health professionals. Increased emphasis has been given to enabling skill acquisition that is relevant and immediately useful through a pedagogical approach. This method of teaching is scalable to all disciplines and adaptable to various forms of instruction including in-person and virtual [22].

While there has been increased emphasis on technology as a means of connection in the academic and professional setting, there is still a lack of resources, perhaps more now than in the past, given the economic challenges that higher educational institutions and society as a whole are facing. Even still, resources are being allocated to determine the best mechanisms for virtual learning and how the school can support those pedagogies. The pandemic has increased the need for SPH to respond with a well-trained workforce armed with skills they can immediately apply [7], as evidenced by the deployment of PH students during the pandemic to support under-resourced local health departments [4,5]. In particular, training students in both technical and professional skill acquisition through applied opportunities is immediately beneficial. As this study indicates, this can be done with similar effectiveness in-person, virtually, or in a hybrid format.

Agencies consistently have limited bandwidth, and students and faculty are still pressed with personal and professional constraints. However, the shift to virtual connection offers an opportunity to engage in new ways that could be streamlined and accessible if leveraged correctly. Allocating resources to this effort is essential [22] to maintaining the caliber of instruction for skill acquisition [6]. Technology for teaching and learning has never been more available as it is now, though it is far from perfect, and additional considerations, such as student engagement, continue to emerge [22]. It is also critical to acknowledge the clear inequities in the technologies available to people, and a learning curve for using them means more training to use technology is essential [27]. Even still, websites and resources that before came at a cost are freely available, and support for using technology is at the forefront of planning and resource allocation. Given the promise of PBT in a virtual environment, as this study shows, leveraging the shift to technology can further enhance pedagogies such as PBT.

This study is one of the first on the effectiveness of achieving student competencies through PBT in a variety of delivery formats. However, there are still some limitations that may affect the generalizability of results. First, the evaluation was conducted on one PBT class focused on certain competencies and deliverables, though a variety of technical and professional skills were assessed. Second, the sample sizes within semesters were low but depended on enrollment for the course and response rates were high across all three semesters. Third, perceptions and attitudes were not controlled for in data collection or analysis and could have been heavily influenced given the context of the pandemic especially during the summer 2020 and fall 2020 semesters. Finally, our evaluation was focused only on immediate post-course feedback in students and does not evaluate follow-up skills in practice or other stakeholder engagement (i.e., collaborating agencies, faculty, and school).

There are many strengths to this evaluation. First, it was conducted by one evaluator unaffiliated with the program who examined this course across time. Second, it examined a variety of perceptions and attitudes related to the utility of PBT and the effect of virtual learning on achieving course competencies, which is a newer area of study. Third, to ensure consistency for the evaluation, the PBT course was delivered by the same faculty in all three semesters with a virtual client and using the same syllabus and assignment guidelines. Finally, collaborating agencies represented a variety of PH agencies, sectors, and issues.

Virtually delivered PBT holds promise and can be as effective as in-person delivery. Future research should examine long-term outcomes of PBT post-semester to ensure it is being used in practice, regardless of delivery mode. In addition, stakeholder engagement, benefits, and challenges should be assessed for the different delivery modes. Virtual learning will remain part of our higher education landscape and the need to have a ready workforce with the technical and professional skills to apply in practice is essential. Redesigning courses is at the forefront of higher education and will become even more relevant as SPH modify and expand their offerings. Virtual learning will remain part of the higher education landscape and should be leveraged in curriculum revisions with evidence that the pedagogy is effective.

## 5. Conclusions

Even when we return to a place where course instruction can safely occur in the classroom, virtual learning is now here to stay, not just in public health, but in all fields. It offers an opportunity to reach more students, accommodate different schedules, utilize various technologies, engage more partners, and broaden the networks in the field. PBT is not only possible to implement in the current learning environment but is essential; it mimics the virtual collaboration and problem-solving that is our new norm. It is a pedagogy that is scalable and can be employed regardless of PH discipline and, in fact, regardless of field of study. As our study shows, it is equally effective delivered in-person, virtually, or hybrid. The widespread utility of PBT is due to the foundation on which it is built—solving current problems for real agencies that have a responsibility to better serve their communities, and in the process, delivering students the competencies required by the course to meet school and accreditation standards and the field-based skills necessary for success. In higher education, there are different ways of delivering skills to make students more workforce-ready and marketable to the field, and this can be done effectively with PBT whether in-person, virtually, or hybrid. Public health is in a prime position to lead this effort, and utilizing pedagogies such as PBT allows us—academics, practitioners, educators, and researchers—to meet the field where it is and usher a new path forward.

## Figures and Tables

**Table 1:** Student Characteristics Post-Course across Three Semesters of PBT Course: In-Person, Virtual, and Hybrid (*n* = 39).

	Fall 2019 In-Person (N = 16)	Summer 2020 Virtual (N = 8)	Fall 2020 Hybrid (N = 15)
	N (%)	N (%)	N (%)
Age			
Under 25	10 (62.5%)	3 (37.5%)	11 (73.3%)
Over 25	6 (23.5%)	5 (62.5%)	4 (26.7%)
Gender			
Female	14 (87.5%)	8 (100%)	12 (80.0%)
Male	2 (12.5%)	0 (0%)	3 (20.0%)
Race/Ethnicity			
Asian	4 (25.0%)	2 (25%)	6 (40.0%)
White/Caucasian	10 (62.5%)	(0%)	6 (40.0%)
Black/African American	0 (0%)	4 (50.0%)	1 (6.7%)
Hispanic	0 (0%)	1 (12.5%)	0 (0%)
Other	1 (6.3%)	0 (0%)	2 (13.3%)
Multiple Other	1 (6.3%)	1 (12.5%)	0 (0%)

**Table 2:** Post-Course Student Skill Acquisition across Three Semesters of PBT Course: In-Person, Virtual, and Hybrid (*n* = 39).

Skill: How Important Was the Use of PBT for You in Acquiring the Following Skills? ^1^	Fall 2019 (In-Person) Mean ^2^ (Std) *n* = 16	Summer 2020 (Virtual) Mean ^2^ (Std) *n* = 8	Fall 2020 (Hybrid) Mean ^2^ (Std) *n* = 15	Virtual vs. In-Person *p*-Value ^3^	Hybrid vs. In-Person *p*-Value ^3^
Consultation techniques	3.63 (0.62)	4.50 (0.76)	3.47 (0.74)	0.0077	0.5746
Conducting a literature review	3.00 (0.89)	3.13 (0.64)	3.20 (0.94)	0.8679	0.5286
Development of performance objects	3.75 (0.45)	4.13 (0.64)	3.53 (0.74)	0.1343	0.5019
Writing for the media	3.75 (0.58)	4.38 (0.52)	3.53 (0.64)	0.0124	0.2424
Development of logic model	3.69 (0.48)	4.13 (0.35)	3.53 (0.64)	0.0426	0.5544
Design of communication plan	3.75 (0.45)	4.50 (0.53)	3.73 (0.46)	0.0037	0.9377
Giving presentations	3.25 (0.77)	4.25 (0.89)	3.33 (0.82)	0.0188	0.7311
Design media executions	3.63 (0.62)	4.63 (0.52)	3.73 (0.59)	0.0012	0.5350
Program evaluations	3.36 (0.84)	4.25 (0.71)	3.20 (0.77)	0.0245	0.5382
Multi-media communication	3.63 (0.62)	4.63 (0.52)	3.47 (0.64)	0.0012	0.4337

^1^ Competencies listed map to the requirements of the course. ^2^ Response options ranged from 1 = not at all important; 2 = somewhat unimportant; 3 = neither important nor unimportant; 4 = somewhat important; 5 = very important. ^3^ *p*-values represent the comparison between in-person and hybrid and in-person and virtual and are calculated at an alpha of 0.05 using the Whitney–Mann Wilcoxon rank test.

**Table 3:** Post-Course Student Attitudes and Beliefs of PBT across Three Semesters: In-Person, Virtual, and Hybrid (*n* = 39).

Statements: ^1^	Fall 2019 (In-Person) Mean ^2^ (Std) *n* = 16	Summer 2020 (Virtual) Mean ^2^ (Std) *n* = 8	Fall 2020 (Hybrid) Mean ^2^ (Std) *n* = 15	Virtual vs. In-Person *p*-Value ^3^	Hybrid vs. In-Person *p*-Value ^3^
I appreciate the utility of the course as PBT more now than when I was enrolled.	4.25 (1.14)	3.75 (1.28)	4.67 (0.82)	0.3583	0.2360
The time invested in taking a PBT course was worth it.	4.93 (0.26)	4.75 (0.46)	4.67 (1.05)	0.2457	0.5501
I would have gained the same knowledge if the course utilized traditional teaching methods instead of PBT.	1.69 (1.20)	2.38 (1.41)	2.00 (0.96)	0.1160	0.1391
I would have gained the same skills if the course utilized traditional teaching methods.	1.60 (1.24)	2.25 (1.49)	1.80 (1.01)	0.1398	0.2452
More MPH courses should utilize PBT.	4.81 (0.40)	4.63 (0.74)	4.64 (1.08)	0.6550	0.8473

^1^ Statements asked for post-course reflections regarding PBT. ^2^ Response options ranged from 1 = strongly disagree; 2 = disagree; 3 = neither agree nor disagree; 4 = agree; 5 = strongly agree. ^3^ *p*-values represent the comparison between in-person and hybrid and in-person and virtual and are calculated at an alpha of 0.05 using the Whitney–Mann Wilcoxon rank test.

**Table 4:** Post-Course Student Perceptions on Workforce Readiness across Three Semesters of PBT Course: In-Person, Virtual, and Hybrid (*n* = 39).

In My Current Work Environment, I Find That Having Worked with a Client through PBT… ^1^	Fall 2019 (In-Person) Mean ^2^ (Std) *n* = 16	Summer 2020 (Virtual) Mean ^2^ (Std) *n* = 8	Fall 2020 (Hybrid) Mean ^2^ (Std) *n* = 15	Virtual vs. In-Person *p*-Value ^3^	Hybrid vs. In-Person *p*-Value ^3^
Helped develop my problem-solving skills	4.70 (0.48)	4.25 (0.71)	4.77 (0.44)	0.1568	0.7445
Enhanced my leadership skills	4.73 (0.47)	4.50 (0.53)	4.21 (0.81)	0.3485	0.0666
Made me more marketable	4.90 (0.32)	4.75 (0.46)	4.57 (1.09)	0.4497	0.4698
Allowed me to acquire skills I could implement in my job	4.60 (0.52)	4.63 (0.52)	4.86 (0.36)	0.9581	0.1722
Enhanced my skills working with a team	4.56 (0.53)	4.50 (0.76)	4.73 (0.46)	1.0000	0.4014
Enhanced my appreciation for the field of public health	4.89 (0.33)	4.63 (0.74)	4.67 (1.05)	0.4688	0.8763
Helped clarify my future plans	4.60 (0.52)	3.63 (1.19)	4.89 (0.33)	0.0609	0.1814
Made me better prepared to enter the workforce	4.64 (0.51)	4.00 (0.76)	4.47 (0.83)	0.0616	0.7841
Resulted in professional networking opportunities I might not have otherwise encountered	4.60 (0.55)	3.38 (0.92)	4.40 (0.97)	0.0324	0.9436

^1^ Statements asked for post-course reflections on workforce readiness as a result of PBT. ^2^ Response options ranged from 1 = strongly disagree; 2 = disagree; 3 = neither agree nor disagree; 4 = agree; 5 = strongly agree. ^3^ *p*-values represent the comparison between in-person and hybrid and in-person and virtual and are calculated at an alpha of 0.05 using the Whitney–Mann Wilcoxon rank test.

**Table 5:** Post-Course Student Assessment of Impacts of Virtual PBT Course on Skill Achievement across Three Semesters: In-Person, Virtual, and Hybrid (*n* = 39).

Please Consider How the Following Were Affected by Participating in SB806 in a Virtual Environment ^1^	Summer 2020 (Virtual) Mean ^2^ (Std) *n* = 8	Fall 2020 (Hybrid) Mean ^2^ (Std) *n* = 15	*p*-Value ^3^
Developing my problem-solving skills	4.25 (0.71)	3.47 (0.74)	0.0232
Enhancing my leadership skills	3.75 (0.71)	3.33 (0.90)	0.2235
Acquiring skills I could implement in my job	3.63 (0.74)	3.46 (0.99)	0.5392
Enhancing my skills working with a team	3.75 (1.04)	3.53 (0.92)	0.6110
Making me better prepared to enter the workforce	3.86 (0.69)	3.67 (0.90)	0.6251
Meeting course expectations/ learning outcomes	3.13 (083)	3.73 (1.10)	0.2147
Producing quality assignments	3.25 (0.89)	3.53 (0.92)	0.4732
Engaging with course activities (lectures, brainstorming sessions, skill building work)	2.75 (0.89)	2.67 (1.35)	0.6544
Interacting productively with the teaching team	3.50 (1.07)	3.40 (1.24)	0.8150
Establishing a personal connection with the teaching team	3.25 (1.04)	2.71 (1.07)	0.1764
Interacting productively with my client	3.13 (0.35)	2.93 (0.80)	0.3201
Establishing a personal connection with my client	2.75 (0.71)	2.60 (1.12)	0.6092
Establishing a personal connection with other students in the course	2.63 (0.92)	2.53 (1.13)	0.6599
Staying motivated to produce my best work	2.88 (0.83)	2.80 (1.26)	0.7883

^1^ Statements asked for impacts of the virtual learning environment on skill development and workforce readiness. ^2^ Response options ranged from 1 = Very negatively impacted by working virtually; 2 = Somewhat negatively impacted by working virtually; 3 = Not impacted; 4 = Somewhat positively impacted by working virtually; 5 = Very positively impacted by working virtually. ^3^ *p*-values represent the comparison between hybrid and virtual and are calculated at an alpha of 0.05 using the Whitney–Mann Wilcoxon rank test.

## Data Availability

The data presented in this study are available on request from the corresponding author. The data are not publicly available due to student confidentiality and privacy regulations.
